# Factors associated with turnover intention among institutional long-term care workers in China: a multi-center cross-sectional study in China

**DOI:** 10.3389/fpubh.2026.1820617

**Published:** 2026-07-03

**Authors:** Baohua Liu, Hongguo Zhou, Shuping Zhou, Meifei Ruan, Lingbo Zhao, Xiaoling Huang, Yan Chen, Julin Yang, Xudong Zhao, Huiyin Lin

**Affiliations:** 1 Department of Elderly Care and Management, School of Health Services and Wellness, Ningbo College of Health Sciences, Ningbo, Zhejiang, China; 2 Dean’s Office, Ningbo College of Health Sciences, Ningbo, Zhejiang, China; 3 Department of Development Planning, Ningbo College of Health Sciences, Ningbo, Zhejiang, China; 4 School of Nursing, Ningbo College of Health Sciences, Ningbo, Zhejiang, China; 5 Department of Organization, Ningbo College of Health Sciences, Ningbo, Zhejiang, China; 6 Continuing Education College, Ningbo College of Health Sciences, Ningbo, Zhejiang, China

**Keywords:** job burnout, long-term care worker, resource, supervisor support, work challenges

## Abstract

**Introduction:**

As population aging accelerates, stabilizing the long-term care workforce has become increasingly important. As a key cognitive precursor of actual turnover behavior, turnover intention provides an important indicator of workforce instability among long-term care workers.

**Objective:**

The aim of this study was to describe turnover intention and explore its associated factors among institutional long-term care workers.

**Methods:**

A cross-sectional self-administered anonymous questionnaire survey was conducted among long-term care workers from 28 older long-term care institutions. Data were collected between November and December 2025 in Zhejiang Province, China. A total of 808 valid samples were included in the study. Hierarchical logistic regression was used to assess the relationship between turnover intention and associated factors. Odds ratios (ORs) and 95% confidence intervals (CIs) for OR were calculated.

**Results:**

Approximately 23.0% of the respondents (186/808) were classified as having high turnover intention. The multivariable logistic regression model (Model 4) showed that, compared with age 35 years or younger, being older than 55 years (OR = 0.619; 95% CI: 0.402–0.954) was negatively associated with high turnover intention. Working in public institutions (OR = 0.487; 95%CI = 0.326–0.728) and having a high level of supervisor support (OR = 0.525; 95%CI = 0.331–0.833) were also negatively associated with high turnover intention. Perceived heavy workload (OR = 2.195; 95%CI = 1.315–3.663), perceived difficulty taking leave (OR = 1.882; 95%CI = 1.184–2.991), higher levels of job burnout (OR = 1.046; 95%CI = 1.028–1.064), and poorer sleep quality (OR = 1.079; 95%CI = 1.014–1.149) showed significant positive associations with high turnover intention.

**Conclusion:**

The turnover intention of institutional long-term care workers is associated with a range of complex factors. Modifiable factors associated with turnover intention, including supervisor support, heavy workload, difficulty taking leave, job burnout, and poor sleep quality, may provide practical targets for human resource management in long-term care institutions. Therefore, managers should consider organizational-level measures to strengthen support for care workers, improve the work environment, and develop retention strategies that may help reduce turnover intention.

## 1 Introduction

The accelerating pace of population aging worldwide, along with the increasing prevalence of age-related conditions and rising disability rates, is imposing significant strain on social care services globally. Meeting the care needs of older adults has become a new topic related to the livelihood security of many countries (1). Compared with global trends, China’s rapidly aging population and its status as the home to the world’s largest older population make the mismatch between care supply and demand particularly prominent and urgent (2, 3). The population of older adults with disabilities and dementia is projected to grow rapidly in the coming decades (4, 5). A growing number of older adults lack access to home care, and thus the responsibility for long-term care is gradually being shifted to society. As a professional setting for meeting complex care demands, especially among older adults with disabilities, institutional long-term care has assumed an increasingly prominent role. By 2023, China had over 400,000 long-term care institutions nationwide housing approximately 8.5 million older adults, and absorbing more than 60% of the country’s long-term care workforce (2). The sustainability and quality of institutional care depend heavily on the stability of this workforce. Although these workers play a pivotal role, their occupational dilemmas and turnover intention in China’s long-term care sector remain insufficiently understood (6).

Long-term care workers in institutional settings face persistent and substantial occupational challenges. Key issues affecting this workforce include understaffing, limited professional qualifications, imbalanced workforce composition, and low workforce stability (7). In addition, non-standard employment is prevalent in the sector, and long-term care workers generally earn less than their counterparts in other health fields (8). The majority of this care workforce comprises rural migrants with low educational levels, predominantly middle aged and older women (9). Their professional and living conditions are particularly concerning. On the one hand, they have long endured long working hours, heavy workloads, and low income, while also facing limited social recognition. On the other hand, they often lack adequate access to professional training and career development support. Long-term care work is commonly stereotyped as feminized, low-paid, unskilled manual labor, which leads to a serious underestimation of its social value (10).

The shortage of long-term care staff is a global issue, characterized by a notably high turnover rate in institutional settings (11, 12). High staff turnover severely compromises an institution’s capacity to deliver high-quality care, promote a healthy and safe working environment, and ensure a reasonable workload (13). Intention to leave is the primary cognitive precursor of actual turnover and has significant explanatory power (14). Turnover intention may better reflect the actual level of organizational management, and it can be mitigated through targeted organizational intervention (6, 15). Employees with high turnover intention may also find it difficult to fully engage in their work, resulting in a decline in work quality (16). Recruiting and training new employees incurs costs and takes time. In the long-term care sector, such intention may further exacerbate workforce shortages and reduce service productivity. Once high turnover intentions manifest as actual departures, the organization not only loses its prior investments in employee development without yielding a return, but also faces disrupted continuity of care and adverse impacts on remaining staff, including diminished morale and intensified workloads. Therefore, to achieve the goal of providing high quality care to older adults, understanding the various factors associated with the intention to leave among long-term care workers is essential.

A clearer understanding of the predictors of turnover intention can inform evidence-based human resource strategies, improve workforce retention, and ultimately enhance the quality, safety, and person-centered care for older residents (17, 18). Studies have demonstrated that the factors associated with turnover intention include age, work tenure, low income, excessive workload, lack of social support, low job satisfaction, exposure to workplace violence, and job burnout (19–21). However, these findings are mainly derived from medical personnel, while research on the associated factors of turnover intention among long-term care workers in institutional settings remains scarce, especially in China (6).

Given the high turnover rate among long-term care workers represents a pervasive worldwide challenge, ensuring workforce stability has become a critical priority in the context of a rapidly aging global population. Therefore, investigating turnover intention and its associated factors among long-term care workers in China may provide valuable insights into workforce retention in long-term care and contribute to addressing the broader global challenge of long-term care workforce turnover.

This study draws on the Conservation of Resources (COR) theory (22), which posits that individuals are motivated to obtain, retain, and protect valued resources. According to COR theory, individual resources are important for understanding work-related outcomes, and negative work events may be viewed as conditions that threaten or deplete individual resources. When individuals face resource loss, insufficient resource replenishment, or the threat of potential resource loss, organizational support may serve as an important contextual resource that helps them cope with work-related demands. In contrast, insufficient resources may be associated with psychological strain and negative emotions, which are theoretically linked to adverse work-related outcomes, including turnover intention (23). The COR theory also suggests that social support and psychological capital may function as strategic resources in stressful work contexts (24). Therefore, guided by the COR theory, the aims of the present study were to investigate turnover intention among long-term care workers in China and further to examine whether factors conceptually related to resource depletion, such as daily working hours, monthly rest days, perceived occupational dilemmas; resource supplementation variables, such as supervisor support, work atmosphere, job satisfaction; and physical and psychological status, such as job burnout, sleep quality were associated with turnover intention.

## 2 Materials and methods

### 2.1 Study design and sample size

The cross-sectional method was used in this study. The data were collected from long-term care workers in institutional settings in Zhejiang Province between November and December 2025. Participating institutions were recruited using a convenience sampling method. We initially contacted the managers of 32 long-term care institutions in Zhejiang Province. After receiving an explanation of the study’s purpose and procedures, 28 institutions agreed to participate. Managers at the participating institutions were then asked to forward the online survey link to the long-term care workers. The inclusion criteria for this study were as follows: (a) directly providing care for older adults in daily work and (b) agreeing to participate. The exclusion criteria were as follows: (a) being in the probationary period and (b) being on leave or rest during the survey period. The purpose of the study and the rights of the participants were clearly stated in the introductory section of the online survey. Participants were informed that completion and submission of the questionnaire would be regarded as providing informed consent. The online survey was anonymous to protect the confidentiality of respondents’ identities. The study was approved by the institutional review committee of the author’s university (application No.: 2023–006). Because the total number of eligible long-term care workers who received the survey invitation was not available, a true response rate could not be calculated.

The survey platform required mandatory responses for all questionnaire items, thereby ensuring no missing data in the final dataset. To ensure response quality, two data quality screening approaches were used. First, two consistency check items were embedded in different parts of the questionnaire to assess whether respondents answered attentively. Questionnaires with inconsistent responses to these items were excluded. Second, responses to Likert-scale measures were screened for patterned responding. Questionnaires were excluded if respondents exhibited obvious straight-line responding within a scale, defined as selecting the same response option for all items in that scale, such as the burnout scale. A total of 980 questionnaires were returned. After data-quality screening, 808 questionnaires were included in the final analysis, yielding a valid questionnaire rate of 82.4% among returned questionnaires.

### 2.2 Measures

#### 2.2.1 Dependent variable

Turnover intention was assessed using two items (25, 26), namely “I do not want to continue working in this organization” and “I do not want to pursue this career any longer.” Each item was measured on a 7-point Likert scale ranging from 1 (completely disagree) to 7 (completely agree). The total score ranged from 2 to 14, with higher scores indicating stronger turnover intention. For the analysis, turnover intention was dichotomized based on the theoretical midpoint of the total score range (i.e., a total score of 8 or higher) (27). The internal consistency of the two-item measure was good in the present sample, with a Cronbach’s alpha of 0.888.

#### 2.2.2 Independent variable

##### 2.2.2.1 Sociodemographic variables

Sociodemographic variables included sex, age, educational background, marital status, residential area, monthly income, and institutional type.

##### 2.2.2.2 Resource depletion variables

Perceived career predicament among Chinese long-term care workers were evaluated via a 9-item binary scale specifically developed for this study. Participants responded to the prompt, “Do you think the following problems exist in your current work?” by indicating “Yes” (coded as 1) or “No” (coded as 0) for each item. The instrument assessed nine specific workplace stressors: low salary, communication difficulties with older adults, lack of public respect, lack of respect from the older adults and their families, unsatisfactory welfare benefits, long-term salary stagnation, heavy workload, difficulty taking leave, and perceived dirtiness of caring for older adults.

Daily working hours and monthly rest days were assessed using two items: “How many hours do you usually work per day?” and “How many days off do you have per month?” Guided by the COR theory, these variables were used to operationalize high-intensity work exposure in terms of work demands and recovery opportunities. Daily working hours were dichotomized at 12 h, with ˃12 h per day indicating long working hours. Monthly rest days were dichotomized at 4 days, with ≤4 rest days per month indicating insufficient rest.

##### 2.2.2.3 Resource supplementation variables

Job satisfaction was evaluated using a single item: “Overall, how satisfied are you with your current job?”, rated on a 5-point Likert scale ranging from 1 (extremely dissatisfied) to 5 (extremely satisfied). Work atmosphere was measured via the item: “How would you describe the current work atmosphere?”, with responses scored from 1 (highly unfriendly) to 5 (highly friendly). Supervisor support was assessed with the item: “If you need help, can you obtain it from your supervisors?”, scored on a 5-point Likert scale (1 = never possible, 5 = always possible). For subsequent analyses, these variables were dichotomized: job satisfaction was categorized into ‘satisfied’ (scores 4–5) versus ‘dissatisfied’ (scores 1–3); work atmosphere into ‘friendly’ (scores 4–5) versus ‘unfriendly’ (scores 1–3); and supervisor support into ‘high’ (scores 4–5) versus ‘low’ (scores 1–3).

##### 2.2.2.4 Physical and mental outcome variables after resource exhaustion

The 15-item Maslach Burnout Service Inventory (MBI-GS) was adopted, including 5 items on emotional exhaustion, 4 items on cynicism, and 6 items on personal accomplishment. Each item is rated on a 7-point Likert scale, ranging from 0 (never) to 6 (very frequent), yielding a total job burnout score of 0 to 90. The Cronbach’s *α* coefficient for the scale was 0.815.

Sleep quality among long-term care workers over the past month was assessed using the Pittsburgh Sleep Quality Index (PSQI). The Chinese version of the PSQI has previously demonstrated good reliability and test–retest validity (28). It comprises 19 items and seven components: sleep duration, sleep efficiency, subjective sleep quality, sleep medication use, sleep latency, disturbances, and daytime dysfunction. The score range for each component is 0 to 3. The total score is calculated by summing the scores for each component and consequently ranges from 0 to 21, with higher scores indicating poorer sleep quality.

### 2.3 Statistical analysis

Skewness, kurtosis tests, and the Q-Q plot were used to examine the normality of the distribution for each continuous data set. According to Kim, for samples larger than 300, an approximate normal distribution can be assumed for variables with absolute values of skewness below 3 and kurtosis below 8 (29). The results showed that the skewness values of each continuous variable ranged from −0.51 to 2.43, and the kurtosis values ranged from −0.94 to 6.91, respectively, which indicates an approximate normal distribution (for details, see Table 1) (29). The Q–Q plot test results indicate that the variables of sleep quality and job burnout do not deviate significantly from a normal distribution.

**Table 1:** Characteristics of respondents (*n* = 808).

Variables	Coding	*n* (%)/mean ± SD	Turnover intention		χ^2^/F	*P*
			High(*N* = 186)	Low(*N* = 622)		
Gender						
Male	1	105(13.0)	22(21.0)	83(79.0)	0.291	0.590
Female	0	703(87.0)	164(23.3)	539(76.7)		
Age						
≤35	1	162(20.0)	57(35.2)	105(64.8)	18.530	**<0.001** ^***^
36 ~ 55	2	460(56.9)	98(21.3)	362(78.7)		
>55	3	186(23.0)	31(16.7)	155(83.3)		
Marital status						
Married	1	626(77.5)	137(21.9)	489(78.1)	2.020	0.155
Unmarried/Divorced/Widowed	0	182(22.5)	49(26.9)	133(73.1)		
Living area						
Urban area	1	241(29.8)	52(21.6)	189(78.4)	0.404	0.525
Rural area	0	567(70.2)	134(23.6)	433(76.4)		
Monthly income						
≥6,000 yuan	1	202(25.0)	44(21.8)	158(78.2)	0.233	0.629
<6,000 yuan	0	606(75.0)	142(23.4)	464(76.6)		
Education background						
College degree or above	1	209(25.9)	67(32.1)	142(67.9)	12.994	**<0.001** ^***^
High school/vocational school and below	0	599(74.1)	119(19.9)	480(80.1)		
Institutional type						
Public institution	1	546(67.6)	106(19.4)	440(80.6)	12.355	**<0.001** ^***^
Private institution	0	262(32.4)	80(30.5)	182(69.5)		
Daily working hours						
>12 h	1	155(19.2)	42(27.1)	113(72.9)	1.799	0.180
≤12 h	0	653(80.8)	144(22.1)	509(77.9)		
Monthly days off						
>4 days per month	1	238(29.5)	58(24.4)	180(75.6)	0.347	0.556
≤4 days per month	0	570(70.5)	128(22.5)	442(77.5)		
Low salary						
Yes	1	408(50.5)	128(31.4)	280(68.6)	32.448	**<0.001** ^***^
No	0	400(49.5)	58(14.5)	342(85.5)		
Communication difficulties with older adults						
Yes	1	249(30.8)	80(32.1)	169(67.9)	16.851	**<0.001** ^***^
No	0	559(69.2)	106(19.0)	453(81.0)		
Lack of public respect						
Yes	1	188(23.3)	69(36.7)	119(63.3)	25.883	**<0.001** ^***^
No	0	620(76.7)	117(18.9)	503(81.1)		
Lack of respect from older adults and their families						
Yes	1	173(21.4)	59(34.1)	114(65.9)	15.262	**<0.001** ^***^
No	0	635(78.6)	127(20.0)	508(80.0)		
Unsatisfactory welfare benefits						
Yes	1	307(38.0)	105(54.2)	202(65.8)	34.937	**<0.001** ^***^
No	0	501(62.0)	81(16.2)	420(83.8)		
Long-term salary stagnation						
Yes	1	412(51.0)	127(30.8)	285(69.2)	28.902	**<0.001** ^***^
No	0	396(49.0)	59(14.9)	337(85.1)		
Heavy workload						
Yes	1	337(41.7)	121(35.9)	216(64.1)	54.166	**<0.001** ^***^
No	0	471(58.3)	65(13.8)	406(86.2)		
Difficulty taking leave						
Yes	1	286(35.4)	108(37.8)	178(62.2)	54.296	**<0.001** ^***^
No	0	522(64.6)	78(14.9)	444(85.1)		
Perceived dirtiness of caring for older adults						
Yes	1	281(34.8)	90(32.0)	191(68.0)	19.731	**<0.001** ^***^
No	0	527(65.2)	96(18.2)	431(81.8)		
Job satisfaction						
Satisfied	1	675(83.5)	140(20.7)	535(79.3)	12.020	0.001^**^
Dissatisfied	0	133(16.5)	46(34.6)	87(65.4)		
Work atmosphere						
Friendly	1	656(81.2)	129(19.7)	527(80.3)	22.152	**<0.001** ^***^
Unfriendly	0	152(18.8)	57(37.5)	95(62.5)		
Supervisor support						
Yes	1	625(77.4)	107(17.1)	518(82.9)	54.204	**<0.001** ^***^
No	0	183(22.6)	79(43.2)	104(56.8)		
Job burnout	–	27.21 ± 13.92	34.47 ± 11.74	25.04 ± 13.79	21.426	**<0.001** ^***^
Sleep quality	–	5.82 ± 3.15	7.05 ± 3.29	5.45 ± 3.02	23.254	**<0.001** ^***^

Bold type denotes *P* < 0.05. **P < 0.01; ***P < 0.001.

Frequency data were described using counts and percentages. Means and standard deviations (SD) were calculated for continuous variables. Univariate comparisons were performed using chi-squared tests and independent samples t-tests. Guided by the conceptual framework of the Conservation of Resources (COR) theory, hierarchical binary logistic regression analyses were performed to examine the predictors of turnover intention through four nested models: Model 1 established the baseline by including demographic variables (age, gender, marital status, residential area, monthly income, educational background, and institution type); Model 2 incorporated resource depletion variables (daily working hours, monthly rest days, and 9 items of perceived career predicament) in addition to Model 1; Model 3 further introduced resource supplementation variables (job satisfaction, work atmosphere, and supervisor support) based on Model 2; and Model 4 added physical and mental outcomes (job burnout and sleep quality) to construct the final full model. Adjusted odds ratios (aORs) and 95% confidence intervals (CIs) for OR were calculated. Statistical analyses were conducted using SPSS version 24.0, and *p* < 0.05 was defined as significant.

## 3 Results

Table 1 presents the characteristics of the respondents (*N* = 808). Approximately 23.0% (186/808) of the respondents reported a high turnover intention. In the total sample, the average age was 46.3 years. The majority of the respondents were female (87.0%), married (77.5%), and held rural household registration (70.2%), had an education level of high school/vocational school or below (74.1%), and reported a monthly income of less than 6,000 yuan (75.0%).

Univariate analysis showed that age, education background, institutional type, job satisfaction, work atmosphere, supervisor support, low salary, communication difficulties with older adults, lack of public respect, lack of respect from older adults and their families, unsatisfactory welfare benefits, long-term salary stagnation, heavy workload, difficulty taking leave, perceived dirtiness of caring for older adults, job burnout, and sleep quality differed significantly between the low and high turnover intention groups (all *p* < 0.05). Detailed information is shown in Table 2.

**Table 2:** Mean, standard deviation, skewness, and kurtosis of continuous variables.

Variables	Total				High turnover intention				Low turnover intention			
	Mean	SD	Skewness	Kurtosis	Mean	SD	Skewness	Kurtosis	Mean	SD	Skewness	Kurtosis
Job burnout	27.21	13.92	−0.21	−0.82	34.47	11.74	−0.51	0.17	25.04	13.79	−0.09	−0.94
Sleep quality	5.82	3.15	0.64	0.27	7.05	3.29	0.63	0.57	5.45	3.01	0.61	0.02

Table 3 shows the results of factors related to turnover intention. All four logistic regression models are significant (*p* < 0. 001), and the ‘−2 Log likelihood’ of each model are given (model 1: 839.575, model 2: 753.737, model 3: 727.287, and model 4: 688.803). Model 4 has the lowest ‘−2 Log likelihood’ and is therefore considered the best fitting model. Model 4 results show that, compared with age 35 years or younger, being older than 55 years (OR = 0.619; 95% CI: 0.402–0.954) was negatively associated with high turnover intention. Working in public institutions (OR = 0.487; 95%CI = 0.326–0.728) and having a high level of supervisor support (OR = 0.525; 95%CI = 0.331–0.833) were also negatively associated with high turnover intention. Perceived heavy workload (OR = 2.195; 95%CI = 1.315–3.663), perceived difficulty taking leave (OR = 1.882; 95%CI = 1.184–2.991), higher levels of job burnout (OR = 1.046; 95%CI = 1.028–1.064), and poorer sleep quality (OR = 1.079; 95%CI = 1.014–1.149) showed significant positive associations with high turnover intention.

**Table 3:** Logistic regression analysis of factors associated with turnover intention.

Variables	Model 1	Model 2	Model 3	Model 4
	OR(95 CI%)	OR(95 CI%)	OR(95 CI%)	OR(95 CI%)
Gender				
Male	0.758(0.449–1.278)	0.678(0.388–1.186)	0.671(0.378–1.192)	0.596(0.327–1.087)
Female				
Age				
≤35	0.568(0.331–0.975)^*^	0.469(0.260–0.846)^*^	0.483(0.262–0.888)^*^	0.546(0.292–1.021)
36 ~ 55	0.446(0.234–0.851)^*^	0.348(0.172–0.702)^**^	0.373(0.181–0.766)^**^	0.429(0.204–0.903)^*^
>55				
Marital status				
Married	1.153(0.731–1.818)	1.094(0.673–1.776)	1.118(0.678–1.844)	1.311(0.780–2.203)
Unmarried/Divorced/Widowed				
Living area				
Urban area	0.812(0.555–1.189)	0.883(0.584–1.333)	0.917(0.603–1.394)	0.868(0.564–1.335)
Rural area				
Monthly income				
≥6,000 yuan	0.844(0.566–1.257)	0.875(0.5671.349)	0.833(0.536–1.296)	0.795(0.500–1.263)
<6,000 yuan below				
Education background				
College degree or above	1.416(0.882–2.272)	1.350(0.789–2.310)	1.497(0.862–2.597)	1.551(0.879–2.738)
High school/vocational school and below				
Institutional type				
Public institution	0.574(0.406–0.811)^**^	0.476(0.326–0.697)^***^	0.500(0.339–0.739)^***^	0.487(0.326–0.728)^***^
Private institution				
Daily working hours				
>12 h	–	1.081(0.671–1.743)	1.146(0.697–1.884)	1.282(0.766–2.143)
≤12 h				
Monthly days off				
>4 days per month	–	0.978(0.630–1.517)	1.008(0.643–1.580)	1.081(0.675–1.731)
≤4 days per month				
Low salary				
Yes	–	1.127(0.696–1.827)	1.143(0.702–1.860)	1.221(0.737–2.023)
No				
Communication difficulties with older adults				
Yes	–	0.938(0.614–1.432)	0.926(0.601–1.425)	0.790(0.506–1.234)
No				
Lack of public respect				
Yes	–	1.664(1.000–2.768)	1.644(0.976–2.769)	1.331(0.776–2.282)
No				
Lack of respect from older adults and their families				
Yes	–	0.823(0.485–1.396)	0.706(0.410–1.217)	0.771(0.443–1.344)
No				
Unsatisfactory welfare benefits				
Yes	–	1.297(0.812–2.074)	1.194(0.736–1.935)	1.159(0.707–1.900)
No				
Long-term salary stagnation				
Yes	–	1.092(0.660–1.807)	0.942(0.562–1.579)	0.846(0.500–1.432)
No				
Heavy workload				
Yes	–	2.277(1.397–3.711)^**^	2.329(1.419–3.823)^**^	2.195(1.315–3.663)^**^
No				
Difficulty taking leave				
Yes	–	2.044(1.310–3.190)^**^	1.912(1.218–2.999)^**^	1.882(1.184–2.991)^**^
No				
Perceived dirtiness of caring for older adults				
Yes	–	0.844(0.531–1.340)	0.869(0.541–1.394)	1.067(0.653–1.744)
No				
Job satisfaction				
Satisfied	–	–	0.843(0.521–1.363)	1.048(0.633–1.733)
Dissatisfied				
Work atmosphere				
Friendly	–	–	0.876(0.544–1.410)	0.936(0.575–1.523)
Unfriendly				
Supervisor support				
Yes	–	–	0.374(0.242–0.578)^***^	0.525(0.331–0.833)^**^
No				
Job burnout	–	–	–	1.046(1.028–1.064)^***^
Sleep quality	–	–	–	1.079(1.014–1.149)^*^
−2 Log likelihood	839.575	753.737	727.287	688.803
Nagelkerke *R*^2^	0.059	0.206	0.248	0.307

^*^P < 0.05, ^**^p < 0.01, ^***^p < 0.001; CI, confidence interval; OR, odds ratio.

Given the conceptual correlation among workplace resources, a formal multicollinearity diagnostic was performed for all explanatory variables entered into the final multivariable model. The variance inflation factors (VIF) for job satisfaction, work atmosphere, and supervisor support were 1.16, 1.31, and 1.39, respectively. The maximum VIF value observed across all variables in Model 4 was 2.02, which is substantially below the conservative threshold of 5.0. These empirical indices confirm that the multi-layered model maintains high statistical stability and that the regression estimates are not distorted by multicollinearity among related workplace environmental factors.

## 4 Discussion

Approximately 23.0% of long-term care workers reported a high turnover intention in the present study, which was lower than those reported in studies from Fujian Province (37.0%) and Canada (58%), but higher than the findings from South Korea (13.4%) (20, 30, 31). The difference may partly reflect the time of the survey. It is possible that long-term care workers with stronger intentions to leave may have already left before data collection, resulting in a lower observed level of turnover intention in the study sample. In addition, such discrepancies may also stem from regional differences and variations in measurement instruments. Future research should develop standardized and comparable measurement tools to strengthen the evidence base for cross contextual comparisons.

Care workers’ turnover intention is often related to multiple factors (30, 32). This study found that younger age, working in private institutions, low supervisor support, perceived heavy workload, difficulty taking leave, higher levels of job burnout, and poor sleep quality were significantly associated with higher odds of high turnover intention. These findings suggest that turnover intention among long-term care workers is shaped by both individual characteristics and workplace conditions. In particular, organizational factors such as institution type, supervisor support, workload, and leave arrangements may play an important role in workforce stability.

Contrary to our expectations, salary, daily working hours, monthly rest days, and job satisfaction were not significantly associated with turnover intention. One possible explanation is that, despite relatively low salaries and long working hours, many long-term care workers may have limited employment alternatives and therefore remain in their current positions. Moreover, often, the salary of long-term care workers is often linked to their working hours and the number of older adults they serve. Higher salaries are also accompanied by heavier workloads and greater care responsibilities. Therefore, single variables of salary level and service hours cannot predict turnover intention. Additionally, a lower willingness to resign may paradoxically reflect limited labor mobility rather than genuine satisfaction with the current job (33). Consistent with this interpretation, studies from the United States and Canada have also found no significant correlation between salary and turnover intention (30, 34). This study found that younger age (≤35 years) is positively associated with high turnover intention, which aligns with previous research among long-term care workers and the broader healthcare personnel (such as nurses) (6, 19, 20). This trend suggests that younger individuals inherently possess greater career mobility and expanded employment alternatives outside the long-term care sector. They may also have higher expectations for salary and job status. Conversely, the lower turnover intention observed among workers aged 55 years and above may reflect their relatively limited alternative labor market opportunities. This actually traps them in their current relatively stable job positions, manifested as a low reported willingness to resign – a sign of giving up rather than being satisfied (33).

In China, long-term care institutions are mainly classified into public and private ones (33). The present study found that care workers in private institutions exhibited significantly higher turnover intention than those in public institutions. Public long-term care institutions are funded by the government, may provide more stable employment conditions and better welfare benefits. Another reasonable explanation is that the public service nature of public institutions may buffer the negative effects of unfavorable working conditions on turnover intentions. This increases existing employees’ tolerance for harsh working conditions and thereby reduces the occurrence of impulsive resignations (33). In contrast, private institutions operate in a more market-oriented manner and focus more on economic benefits. This may contribute to greater job pressure and lower occupational security among care workers, resulting in higher turnover intention.

In this study, long-term care workers who could perceived high supervisor support when needed were less likely to report high turnover intention. This finding was supported by studies conducted in the United States (35) and Bangladesh (36). When long-term care workers feel a lack of supervisor support, they are more likely to report high intentions to resign. Supervisor support contributes to long-term care workers’ well-being and creates a healthy work environment that is conducive to staff retention (37). Employees who perceive supervisor support are more likely to adopt positive work behaviors and less likely to experience negative work outcomes, such as a desire to resign (37, 38). In the management of long-term care institutions, supervisor support and opportunities for workers to voice work related concerns are therefore essential.

In this study, heavy workload and difficulty taking leave were identified as two self-perceived work challenges faced by long-term care workers, and were significantly associated with high turnover intention. Nearly 20% of the participants reported working more than 12 h per day. Although working hours were not identified as an associated factor for turnover intention in this study, a possible reason is that overtime may increase employees’ income, thereby partially buffering their willingness to resign. Nevertheless, this finding suggests that extended working hours remain an important aspect of working conditions in long-term care institutions. According to the COR theory, individual resources are considered important for understanding job-related outcomes. From this perspective, heavy workload may be interpreted as a potential resource-consuming condition that is associated with adverse work-related outcomes, heavy workload may represent a condition of resource depletion, while difficulty taking leave may limit workers’ opportunities for recovery. Both may therefore contribute to adverse work-related outcomes, including turnover intention. Previous studies have also suggested that the inability of long-term care workers to take unpaid leave may contribute to unavoidable turnover (7, 39). A qualitative study found that older adults’ reluctance to be care by substitute workers and managers’ discouragement of rest breaks were important reasons for care workers’ turnover (7). Therefore, long-term care institutions should consider improving staffing and shift arrangements, protecting workers’ rights to rest and leave, and providing adequate compensation or welfare benefits to reduce turnover intention.

Burnout has been widely studied among nurses and other healthcare workers with previous evidence showing its close relationship with turnover intention (40). Consistent with previous research, job burnout was significantly associated with high turnover intention in this study. Burnout is extremely common among long-term care workers (41). Within the COR framework, job burnout can be understood as reflecting the depletion of employees’ emotional, cognitive, and physical energy resources (42). Long-term care workers often face demanding care tasks, physical strain, shift work, and staffing shortages and limited social recognition, all of which may increase the risk of burnout (43). This finding suggests that addressing burnout may be an important component of workforce retention strategies in long-term care institutions.

Poor sleep quality may contribute to physical fatigue, lack of concentration, weakened judgment, and emotional exhaustion (44). In the context of demanding care tasks and potentially irregular work schedules, poor sleep quality may intensify long-term care workers’ perceived workload and make them more likely to consider leaving their jobs. Sleep-related fatigue and cognitive impairment may also increase the risk of errors at work and reduce work efficiency (45). In such a physically and mentally exhausted state, long-term care workers may find it difficult to effectively cope with the high physical and emotional demands of work, thereby increasing turnover intention.

This study contributes to the literature by examining multiple individual, organizational, and work-related factors associated with turnover intention among long-term care workers in China. Despite these strengths, several limitations should be acknowledged. First, the cross-sectional design precludes inferences about temporal precedence or causal relationships. Although guided by the Conservation of Resources framework, the single-wave data captured only static associations; future longitudinal or multi-wave studies are needed to examine dynamic processes over time. Second, convenience sampling of institutions from a single Chinese province, together with survey distribution through institutional managers, may have introduced selection bias at both the institutional and individual levels, limiting the generalizability of the findings. In addition, because the total number of eligible workers who received the survey invitation was unknown, a true response rate could not be calculated. Third, although the two-item turnover intention measure was adapted from previous studies and showed high internal consistency in this sample, its brevity may limit the extent to which it captures the full complexity of turnover intention. In addition, several explanatory variables were assessed using single-item measures and subsequently dichotomized. Although this pragmatic approach was adopted to accommodate the high workload and varying literacy levels of institutional care workers, it may have reduced statistical power and obscured more nuanced gradients. Future studies could use more comprehensive validated scales and more refined measurement approaches to assess turnover intention and workplace factors in greater detail. Fourth, due to the completely anonymous nature of the online survey, institutional identifiers were not recorded; therefore, we could not account for potential clustering within the 28 institutions using multilevel modeling. This limitation should be addressed in future studies.

## 5 Conclusion

The turnover intention among institutional long-term care workers in China is associated with a range of complex factors. Modifiable factors associated with turnover intention, including supervisor support, heavy workload, difficulty taking leave, job burnout, and poor sleep quality, may provide practical targets for human resource management in long-term care institutions. Therefore, managers should consider organizational-level measures to strengthen support for care workers, improve the work environment, and develop retention strategies that may help reduce turnover intention.

## Data Availability

Requests to access these datasets should be directed to liubaohuawoshi@163.com.
